# Supplementary material for: Effect of Geographic Regions on the Flavor Quality and Non-Volatile Compounds of Chinese Matcha
Source: Foods. 2025 Jan 2;14(1):97. doi: 10.3390/foods14010097 (PMC11720590; doi:10.3390/foods14010097)
Supplement: Supplementary file 1 [file foods-14-00097-s001.zip › foods-3336403-supplementary.pdf]

Supplementary figure

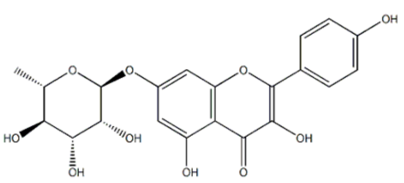

Kaempferol-7-O-rhamnoside

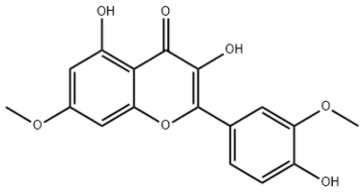

3,7-Di-O-methylquercetin

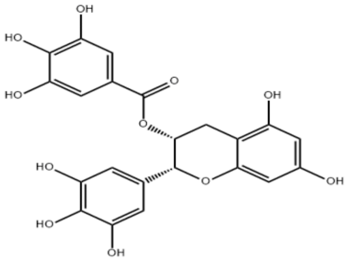

Epigallocatechin gallate

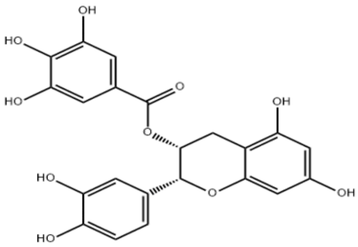

Epicatechin gallate

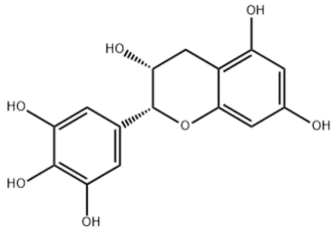

Epigallocatechin

Figure S1. 2D structure of kaempferol-7-O-rhamnoside, 3,7-Di-O-methylquercetin, epigallocatechin gallate, epicatechin gallate, epigallocatechin
